# Can We Improve Patient Safety?

**DOI:** 10.3389/fped.2014.00098

**Published:** 2014-09-18

**Authors:** Martin Thomas Corbally

**Affiliations:** ^1^King Hamad University Hospital, Busatein, Bahrain; ^2^Department of Surgery, Royal College of Surgeons in Ireland – Medical University of Bahrain, Busatein, Bahrain

**Keywords:** patient safety, WHO Surgical Pause/Time Out, parental involvement in Surgical Pause/Time Out, surgeon step back and confirm

## Abstract

Despite greater awareness of patient safety issues especially in the operating room and the widespread implementation of surgical time out World Health Organization (WHO), errors, especially wrong site surgery, continue. Most such errors are due to lapses in communication where decision makers fail to consult or confirm operative findings but worryingly where parental concerns over the planned procedure are ignored or not followed through. The WHO Surgical Pause/Time Out aims to capture these errors and prevent them, but the combination of human error and complex hospital environments can overwhelm even robust safety structures and simple common sense. Parents are the ultimate repository of information on their child’s condition and planned surgery but are traditionally excluded from the process of Surgical Pause and Time Out, perhaps to avoid additional stress. In addition, surgeons, like pilots, are subject to the phenomenon of “plan–continue–fail” with potentially disastrous outcomes. If we wish to improve patient safety during surgery and avoid wrong site errors then we must include parents in the Surgical Pause/Time Out. A recent pilot study has shown that neither staff nor parents found it added to their stress, but, moreover, 100% of parents considered that it should be a mandatory component of the Surgical Pause nor does it add to the stress of surgery. Surgeons should be required to confirm that the planned procedure is in keeping with the operative findings especially in extirpative surgery and this “step back” should be incorporated into the standard Surgical Pause. It is clear that we must improve patient safety further and these simple measures should add to that potential.

Surgical intervention continues to carry a significant and serious risk of complications not associated with the primary disease itself but notably due to preventable human error or wrong site surgery (WSS). Various papers have highlighted an often previously ignored aspect of clinical practice that error can and does occur with serious negative consequences for both the patient and the clinicians involved. Human error and problems with communication are the main cause of such error often compounded by complex clinical environments that can exceed normal individual human performance (1, 2).

While awareness and moreover an acceptance that any individual or practice may be at risk of error has gained slow and sometimes reluctant acceptance, the reported estimates of risk in surgery remain unacceptably high. It is estimated that 5–10 incorrect procedures are performed daily in the USA with a 1% risk of overall mortality. When considered against the background of nearly 234 million operations performed worldwide each year, it is clearly an unacceptable risk (1, 2). Overall, the incidence of major complications during surgery, including WSS, appears to vary between 3 and 22%.

It is clear that when humans interact in complex environments such as hospitals, there will always be a potential for risk or error. This should not detract from our efforts to recognize the potential for error in clinical systems and to insert better, more robust obstacles, and measures to prevent them. Much of what we know today about clinical error has been derived from work done by the Joint Commission on Accreditation of Healthcare Organizations (now called the Joint Commission International-JCI) and the World Health Organization (WHO) (3, 4). Both organizations recognized that irrespective of advances in science and technology, patients were still at significant risk from largely preventable human factors. Recognition of these factors also stressed that the solution to these problems was not punitive but rather should be based on an analysis of events and implementation of more robust safeguards to prevent re-occurrence. Much important insight into human error has been provided by the airline industry that has long recognized and valued the critical importance of human error in airline disasters. However, there has been a slow uptake of the basic concepts of these costly lessons in surgical practice and the incidence of medical error as reported above may in fact be an underestimate. Mandatory reporting structures that report all adverse incidents are essential but not yet widely applied.

The JCI and WHO have introduced safety checks aimed at improving the safety of patients undergoing surgery mostly to confirm the essential aspect of correct patient, correct procedure, and correct side. The process of “Time Out/Surgical Pause” is a series of objective checks to improve patient safety and empower all staff to raise concerns but despite this, error continues in practice. What can be done to further protect the patient? In pediatric surgery, it is common for a parent to be present at anesthetic induction, but they are not typically included in the Surgical Pause. Opposition to their involvement at this stage appears to be a concern that it may add to their distress and anxiety and interestingly to that of theater staff themselves. A recent prospective pilot study has indicated that 100% of theater staff and parents consider that it should be a mandatory part of Time Out/Surgical Pause and that it does not add to anyone’s stress or anxiety (5).

Even with this added barrier in place, it is still possible that surgeons may continue on a “plan–continue–fail” action, which can culminate in WSS. The airline industry has recognized this potential and has installed effective barriers to this action so that any member of the cockpit staff may question a decision and call attention to their concern. This concept needs greater development in clinical practice since the issues of ultimate responsibility and clinical autonomy are less separate and defined as in the airline industry. It does, however, suggest that an additional safety layer should be added to the WHO check list to ensure that in operative situations of extirpative surgery, a final on table “Step Back” stage must be performed where the operating surgeon confirms that the pre-operative diagnosis match the on-table operative findings (6).

It is clear that clinical error will continue to occur wherever humans interact with each other in complex and stressful environments such as the operating theater. Traditional barriers that are considered to be robust can fail to halt progression to an undesirable and tragic outcome. Most major errors are a series of small ones where deficits in communication combine with assumptions that problems or concerns will be dealt with by the system or by some other team member. Acceptance and implementation of team-based responsibilities with team briefings, a Time Out/Surgical Pause that includes parental involvement improves communication and can help prevent WSS. In addition, surgeons must be encouraged to confirm, in extirpative surgery that the operative findings are in keeping with the planned surgical procedure.

Vigilance works but can always be improved on.

## Conflict of Interest Statement

The author declares that the research was conducted in the absence of any commercial or financial relationships that could be construed as a potential conflict of interest.
